# Astrocytic Ca^2+^ signals are required for the functional integrity of tripartite synapses

**DOI:** 10.1186/1756-6606-6-6

**Published:** 2013-01-28

**Authors:** Mika Tanaka, Pei-Yu Shih, Hiroshi Gomi, Takamasa Yoshida, Junichi Nakai, Reiko Ando, Teiichi Furuichi, Katsuhiko Mikoshiba, Alexey Semyanov, Shigeyoshi Itohara

**Affiliations:** 1 RIKEN Brain Science Institute, 2-1 Hirosawa, Wako, 351-0198, Japan; 2 College of Bioresource Sciences, Nihon University, 1866 Kameino, Fujisawa, Kanagawa, 252-0813, Japan; 3 Saitama University Brain Science Institute, 255 Shimo-Okubo, Sakura-ku, Saitama, 338-8570, Japan; 4 ICORP-Sorst, JST, 4-1-8 Honcho, Kawaguchi, 332-0012, Japan; 5 Present address: Department of Applied Biological Science, Faculty of Science and Technology, Tokyo University of Science, 2641 Yamazaki, Noda, Chiba, 278-8510, Japan

**Keywords:** Astrocyte, Calcium, Synapse, Neuron-glia interaction

## Abstract

**Background:**

Neuronal activity alters calcium ion (Ca^2+^) dynamics in astrocytes, but the physiologic relevance of these changes is controversial. To examine this issue further, we generated an inducible transgenic mouse model in which the expression of an inositol 1,4,5-trisphosphate absorbent, “IP_3_ sponge”, attenuates astrocytic Ca^2+^ signaling.

**Results:**

Attenuated Ca^2+^ activity correlated with reduced astrocytic coverage of asymmetric synapses in the hippocampal CA1 region in these animals. The decreased astrocytic ‘protection’ of the synapses facilitated glutamate ‘spillover’, which was reflected by prolonged glutamate transporter currents in *stratum radiatum* astrocytes and enhanced N-methyl-D-aspartate receptor currents in CA1 pyramidal neurons in response to burst stimulation. These mice also exhibited behavioral impairments in spatial reference memory and remote contextual fear memory, in which hippocampal circuits are involved.

**Conclusions:**

Our findings suggest that IP_3_-mediated astrocytic Ca^2+^ signaling correlates with the formation of functional tripartite synapses in the hippocampus.

## Background

Astrocytes exhibit dynamic Ca^2+^ mobilization via an inositol 1,4,5-trisphosphate (IP_3_)-induced Ca^2+^ release (IICR)-dependent mechanism [1,2]. The role of astrocytic Ca^2+^ dynamics, however, has been debated over the last decade [3]. Some studies report intact hippocampal short-and long-term plasticity in situ in IP_3_ receptor (IP_3_R)-type 2 knockout mice, in which hippocampal astrocytes completely lack IICR [4,5], whereas others report that these mice show no cholinergic-induced long-term potentiation (LTP) in vivo [6,7]. In situ evidence also indicates that hippocampal LTP depends on D-serine release from astrocytes under the control of astrocytic Ca^2+^ signaling [8]. A number of reports describe spontaneous or stimulation-evoked Ca^2+^ activity in astrocytes in vivo [9-12], but still very little is known about the in vivo significance of astrocytic Ca^2+^ mobilization [6,7], especially in higher brain functions such as learning and memory. In the present study, we generated a new mouse model in which astrocytic IICR is attenuated, and examined the role of astrocytic Ca^2+^ signaling at the level of both the tripartite synapse and behavior.

## Results

### Attenuated agonist-evoked IICR in astrocytes by IP_3_ buffering with a glutathione-S-transferase (GST)-IP_3_ sponge

**Figure 1:**
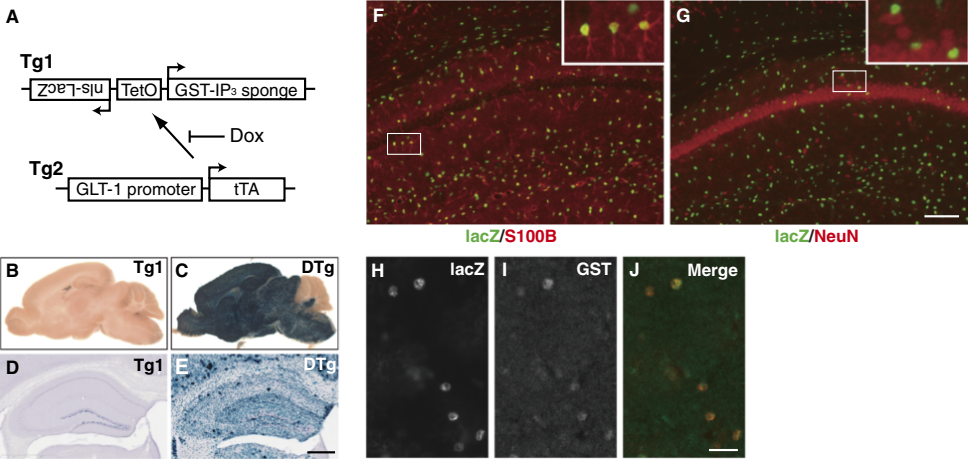
**Astrocyte-specific expression of the GST-IP**_**3**_**sponge.** (**A**) *Tg1*, *Tg2* mouse lines were generated. In the DTg mice, expression of the GST-IP_3_sponge and nls-lacZ is induced under the control of TetO, but can be suppressed by Dox. (**B**, **C**) Whole mount lacZ staining of sagittal brains of *Tg1* (**B**) and DTg (**C**) mice. (**D**, **E**) LacZ staining of coronal hippocampal slices of *Tg1* (**D**) and DTg (**E**) mice. The lacZ expression level in *Tg1* was very low (**B**), but hippocampal dentate gyrus cells showed some leakage (**D**). Scale bar, 0.5 mm. (**F**, **G**) Double immunofluorescence analysis of hippocampal CA1 of DTg mice using antibodies to lacZ, S100B, and NeuN. Insets are magnified images. Scale bar, 100 μm. (**H – J**) Double immunofluorescence analysis using antibodies to lacZ and GST. Scale bar, 25 μm.

To investigate the in vivo role of Ca^2+^ dynamics in astrocytes, we generated two transgenic mouse lines (Figure 1A) in which IICR was attenuated by the expression of an IP_3_ absorbent “IP_3_ sponge” [13] in an astrocyte-specific and temporally controlled manner. In the *Tg1:Tg2* double transgenic (DTg) mice, lacZ reporter expression was efficiently induced in broad brain areas except for the cerebellum (Figure 1, B and C). No gross histologic abnormalities were observed in the brains of the DTg mice. Importantly, lacZ expression was detected in the majority of astrocytes in the DTg mouse hippocampus (Figure 1, D and E). Double immunolabeling revealed that lacZ induction was restricted to the astrocytes (S100B-positive and NeuN-negative cells; Figure 1F and G, Additional file 1: Table S1). In several brain areas, including the hippocampal CA1 and dentate gyrus, 80% to 90% of the S100B-positive cells were lacZ-positive (Additional file 1: Table S1). In addition, the numbers of S100B-positive cells in these brain areas were not significantly different between WT and DTg (Additional file 2: Figure S1). These findings suggest that our system was suitable for astrocyte-specific gene induction in the brain.

We then examined whether the GST-fused IP_3_ sponge was expressed in the same manner as lacZ in the DTg mice. Double immunolabeling detected the GST-IP_3_ sponge in lacZ-positive astrocytes (Figure 1, H to J). The GST-IP_3_ sponge was also detected by glutathione-trapping in the brains of the DTg mice (Additional file 3: Figure S2A). Quantification of the GST-IP_3_ sponge in the brain (Additional file 3: Figure S2, B to E) revealed that GST-IP_3_ sponge expression could be repressed by doxycycline (Dox) treatment (25 μg/ml in the drinking water) initiated either before birth (indicated as “Dox”) or at 1 mo of age (indicated as “Dox*”). Each astrocyte in the DTg mice was estimated to contain approximately 3800 GST-IP_3_ sponge molecules (see Additional file 4: Additional Methods). When transgene expression was inhibited in DTg mice by Dox administration initiated before birth, the induced expression of lacZ did not reach the same level as that in DTg mice without Dox treatment, even 8 weeks after Dox withdrawal (data not shown). Thus, in the following experiments, we used DTg mice without Dox treatment to obtain maximal expression of the IP_3_ sponge for attenuation of astrocytic IICR.

**Figure 2:**
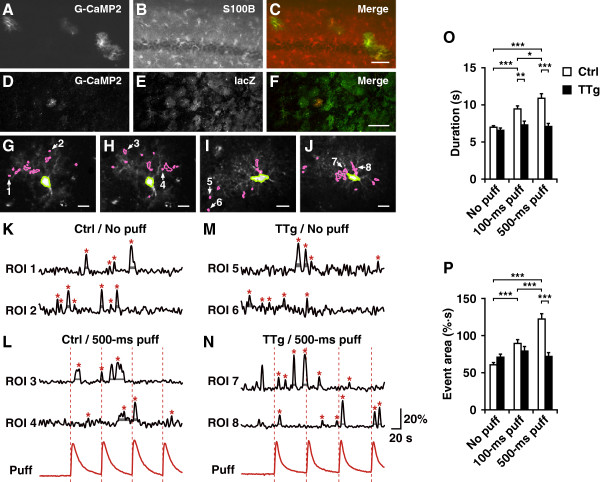
**Ca**^**2+**^**imaging with G-CaMP2.** (**A – C**) Double immunofluorescence analysis of hippocampal CA1 region from *tetO-G-CaMP2:Tg2* (Ctrl) mice using antibodies to G-CaMP2 and S100B. Scale bar, 50 μm. (**D – F**) Confocal images of double immunofluorescence analysis of hippocampal CA1 of *Tg1:tetO-G-CaMP2:Tg2* (TTg) mice using antibodies to G-CaMP2 and lacZ. Scale bar, 100 μm. (**G – J**) Two-photon images of control (Ctrl) and TTg astrocytes with G-CaMP2 fluorescence. Magenta areas are regions of interest (ROIs) in processes that showed changes in fluorescence over time. Green areas indicate soma. (**G**) and (**H**) are the same Ctrl astrocyte and (**I**) and (**J**) are the same TTg astrocyte, but under no-puff (**G**) and (**I**) and 500-ms puff (**H**) and (**J**) conditions. Scale bar, 10 μm. (**K – N**) Ca^2+^ traces in the ROIs indicated by arrows and the numbers in (**G** – **J**). Red traces in (**L**) and (**N**) show changes in the fluorescence induced by 500-ms puff application of Alexa594 with DHPG. Vertical dotted lines in red indicate the onset of puff stimulation. Red asterisks indicate peaks of each Ca^2+^ event. Vertical and horizontal ranges of gray bands below the asterisks represent the range from the baseline + 1SD to + 2SD and the event duration, respectively. Peaks whose duration could not be identified were excluded from the events. Vertical and horizontal scale bars, respectively, indicate 20% (Δ*F*/*F*0) and 20 s. (**O, P**) Mean (±SEM) duration and mean areas under the curve, respectively, of individual events under no puff, 100-ms puff, and 500-ms puff conditions in Ctrl and TTg groups. A significant increase upon stimulation was observed only in the Ctrl group. *P < 0.05, **P < 0.01, ***P < 0.001, with Bonferroni’s correction for multiple comparisons.

We next investigated the effects of GST-IP_3_ sponge expression on Ca^2+^ dynamics in astrocytes in situ. For Ca^2+^ imaging, we used the *tetO-G-CaMP2* transgenic line [14]. Double immunolabeling revealed that the induction of G-CaMP2 expression in the hippocampal CA1 region of the *tetO-G-CaMP2:Tg2* (control, Ctrl) mice was restricted to S100B-positive astrocytes (Figure 2, A to C). We also detected G-CaMP2 expression in lacZ-positive astrocytes in the hippocampal CA1 of *Tg1:tetO-G-CaMP2:Tg2* (triple transgenic, TTg) mice (Figure 2, D to F). The population of G-CaMP2 positive astrocytes was inexplicably restricted by *tetO-G-CaMP2* compared with *Tg1*, presumably due to a positional effect [15-17].

Activation of Gq-protein coupled receptors triggers IICR in astrocytes [4,18]. To examine whether the agonist-evoked IICR is affected by GST-IP_3_ sponge expression, S-3,5-dihydroxyphenylglycine (DHPG), a selective metabotropic glutamate receptor agonist, was puff-applied to CA1 astrocytes expressing G-CaMP2 in hippocampal slices from either control or TTg mice. Ca^2+^ imaging of the astrocytic processes (Figure 2, G to N) revealed that the duration (Figure 2O) and area under the curve (Figure 2P) of Ca^2+^ events of the DHPG-evoked Ca^2+^ responses were both significantly attenuated in the TTg mice (*F*_5,3418_ = 19.95, *P* < 0.001, two-way ANOVA for Figure 2O; *F*_5,3418_ = 22.79, *P* < 0.001, two-way ANOVA for Figure 2P; number of cells, 13 for each genotype; number of animals, 5 for Ctrl and 4 for TTg; number of events, see Additional file 5: Table S2). Baseline Ca^2+^ activity without glutamate puffs was not significantly altered in TTg mice (Figure 2, O and P, No puff). These findings indicate that agonist-evoked IICRs in situ were significantly attenuated by GST-IP_3_ sponge expression in astrocytes.

### Reduced synaptic coverage by astrocytic processes in DTg mice

**Figure 3:**
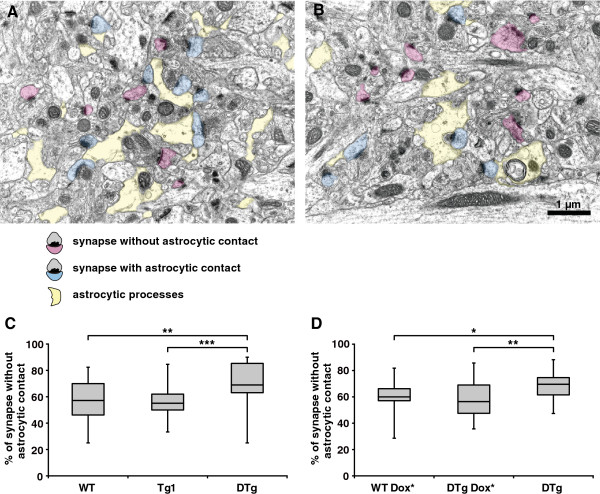
**Astrocytic coverage of asymmetric synapses in DTg mice.** (**A, B**) Electron micrographs of the CA1 *stratum radiatum* of the hippocampus of WT (**A**) and DTg mice (**B**). Colors indicate the classification of astrocytic coverage of asymmetric synapses: pink, synapses without astrocytic contact; blue, synapses with astrocytic contact; yellow, astrocytic processes. (**C**, **D**) Box-and-whisker plots showing pooled data for percent of synapses without astrocytic contact (each box has lines at the lower quartile, median, and upper quartile values, and whiskers indicating the range). Percent of synapses without astrocytic contact was significantly increased in DTg mice [*P =* 0.001, Kruskal Wallis test for 3 groups] (**C**), but was comparable to WT after Dox treatment starting at 1 mo of age (Dox*) [*P =* 0.012, Kruskal Wallis test for 3 groups] (**D**). *P < 0.05, **P < 0.01, ***P < 0.001, two-tailed Mann–Whitney *U* test.

Ca^2+^ activity regulates the morphologic plasticity of growth cones [19]. The link between IICR and the plasticity of the processes has not been established in astrocytes. Thus, we examined the effect of decreased Ca^2+^ activity in DTg mice on the structure of tripartite synapses, which comprise pre- and postsynaptic elements of the synapse and associated astrocytic processes [20,21]. Electron micrographs of the CA1 *stratum radiatum* in the dorsal hippocampus (Figure 3, A and B) revealed reduced astrocytic coverage of the asymmetric synapses in DTg mice. DTg mice had a significantly higher number of asymmetric synapses without astrocytic contact compared to WT and Tg1 (Figure 3C, *P* = 0.001, Kruskal Wallis test for 3 groups; WT vs DTg, *P =* 0.004, Tg1 vs DTg*, P* < 0.0001, two-tailed Mann–Whitney *U* test). Further, the percentage of asymmetric synapses without astrocytic contact in DTg mice treated with Dox beginning at 1 mo of age was comparable to that in WT mice (Figure 3D, *P* = 0.012, Kruskal Wallis test for 3 groups; WT Dox* vs DTg Dox*, *P =* 0.542, two-tailed Mann–Whitney *U* test), suggesting that GST-IP_3_ sponge expression in astrocytes after development led to reduced astrocytic coverage of the synapses in DTg mice. Synaptic density did not significantly differ between genotypes (0.360 ± 0.018 no./ μm^2^ for WT, 0.359 ± 0.017 for *Tg1*, 0.355 ± 0.016 no./ μm^2^ for DTg; *F*_2,57_ = 0.017, *P* = 0.983, non-repeated measures ANOVA). These data suggest that IP_3_-mediated Ca^2+^ signals in astrocytes regulate tripartite synaptic structures.

### Enhanced glutamate spillover at CA1-CA3 synapses in DTg mice

**Figure 4:**
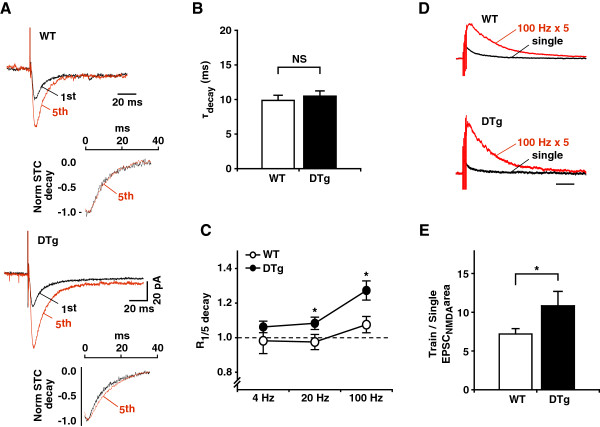
**Enhanced glutamate spillover in DTg mice.** (**A**) STCs elicited by a single stimulus (black trace) and subtracted traces showing responses to the 5^th^stimulus (red trace). Insets: STCs are normalized and aligned so that the peaks are superimposed to facilitate comparison of decay. (**B**) τ_decay_ of STC was not significantly different between WT and DTg mice. n (number of cells) = 16, N (number of animals) = 5 for WT, n = 14, N = 4 for DTg. (**C**) Frequency dependence of R_1/5 decay_ in WT and DTg mice. n = 5, N = 1 for WT, n = 5, N = 2 for DTg. (**D**) Representative NMDA currents recorded from CA1 pyramidal neurons in response to a single stimulus (black trace) and trains of stimuli (5 stimuli, 100 Hz, red trace) at +40 mV. Traces induced by 5 stimuli are normalized to the peak amplitude induced by a single stimulus. (**E**) The ratio of total charge transfer by NMDA receptors (train/single EPSC_NMDA_ area) induced by trains of stimuli to a single stimulus was increased in DTg mice. n = 8, N = 3 for WT, n = 8, N = 4 for DTg. *P < 0.05, unpaired *t* test. The values on the graphs represent mean ± SEM.

Reduced astrocytic coverage of the synapses could lead to increased glutamate spillover and, subsequently, prolonged glutamate clearance by hippocampal astrocytes. The glutamate clearance time-course could be derived from the kinetics of stimulus-induced transporter currents (STCs) in the astrocytes [22]. Therefore, we compared the kinetics of facilitated STCs in hippocampal CA1 *stratum radiatum* astrocytes in response to stimulation of the Schaffer collaterals (Figure 4A). The 20% to 80% rise time (t_rise_, *P* = 0.32, unpaired *t* test) and the decay time constant (τ_decay_) of STCs produced by a single stimulus did not significantly differ between WT and DTg astrocytes (Figure 4B, τ_decay_, *P* = 0.29, unpaired *t* test). This result, in combination with the immunohistochemistry findings of unaltered glial glutamate transporter −1 (GLT-1) expression levels in DTg mice (Additional file 6: Figure S3), suggests that baseline glutamate release and clearance were not affected in DTg mice. To induce glutamate spillover, we delivered high-frequency stimulation and examined the kinetics of STCs induced by burst stimulation (5 stimuli) with increasing frequency (4 Hz, 20 Hz, 100 Hz) in DTg astrocytes. To describe how the transporter current kinetics change with an increased number of stimuli, we calculated the ratio between t_rise_ of isolated STCs to the 5^th^ stimulus and t_rise_ of STCs to a single stimulus (R_1/5 rise_), and the ratio of τ_decay_ in the same way (R_1/5 decay_). The R_1/5 rise_ did not differ between genotypes at any stimulation frequency examined (4 Hz, *P* = 0.32; 20 Hz, *P* = 0.47; 100 Hz, *P* = 0.09, unpaired *t* test). In contrast, the R_1/5 decay_ was significantly larger in DTg astrocytes at higher stimulation frequencies (Figure 4C, 4 Hz, *P* = 0.175; 20 Hz, *P* = 0.046; 100 Hz, *P* = 0.013, unpaired *t* test). The slowdown of decay with an unchanged rise time of STC in DTg astrocytes at high-frequency stimulation is consistent with increased glutamate spillover. Reflecting the insufficient clearance of glutamate, the STC amplitude induced by burst stimulation showed a larger frequency-dependent increase in DTg than in WT astrocytes (Figure 4A).

Changes in glutamate spillover could affect synaptic transmission and presynaptic release probability by acting on presynaptic receptors in hippocampal synapses. We detected no significant differences, however, in the input–output relationship (Additional file 7: Figure S4A) and in the paired-pulse facilitation (Additional file 7: Figure S4B) of field excitatory postsynaptic potentials in the hippocampal CA1 region of DTg mice, suggesting a lack of presynaptic changes with these stimulation protocols. Glutamate spillover during high-frequency burst stimulation could recruit additional synaptic and extrasynaptic N-methyl-D-aspartate receptors (NMDARs) [23,24]. This process is limited by perisynaptic transporters and could thus be enhanced in DTg mice. Therefore, we compared EPSCs mediated by NMDARs (EPSC_NMDAR_) in response to a single stimulus and a train of 5 stimuli at 100 Hz (Figure 4D). Consistent with enhanced glutamate spillover, EPSC_NMDAR_ induced by high frequency stimulation normalized to a single stimulus EPSC_NMDAR_ was enhanced in DTg mice (Figure 4E, *P* = 0.045, unpaired *t* test).

### Impaired spatial reference memory and remote contextual fear memory in DTg mice

**Figure 5:**
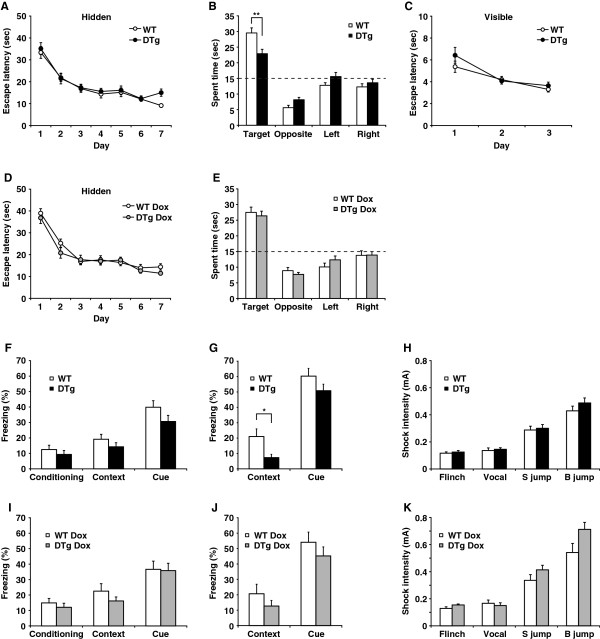
**Behavioral impairments in DTg mice.** (**A – C**) Morris water maze test. Acquisition rates in the hidden platform task did not significantly differ between DTg mice and WT mice (**A**, N = 23 for WT, N = 24 for DTg), but DTg mice spent significantly less time in the target quadrant during the probe test than did WT mice (**B**). Dashed line indicates chance level. No difference was observed between genotypes in the visible platform task (**C**). (**D**, **E**) DTg mice treated with Dox (DTg Dox) were not impaired in the hidden platform task (**D**) or probe test (**E**, N = 24/genotype). (**F – H**) Fear-conditioning and footshock sensitivity test. (**F**, **G**) Each column represents mean percent time freezing during the last 2-min recording period for conditioning, 5-min recording for contextual test, and the last 3-min recording period for cued test. DTg mice showed normal contextual and cued fear memory at 1 d and 2 d after conditioning, respectively (**F**). In contrast, DTg mice exhibited significantly reduced remote contextual fear memory at 30 d after conditioning (**G**), whereas they showed normal remote cued fear memory (**G**) and footshock sensitivity (**H**, N = 12 for each genotype). (**I – K**) DTg mice treated with Dox were not impaired in recent (**I**) or remote (**J**) fear memory, or footshock sensitivity (**K**, N = 12 for each genotype). *P < 0.05, **P < 0.01, unpaired *t* test. Results are provided as mean ± SEM. Flinch, animal flinched; Vocal, animal vocalized; S jump, small jump; B jump, big jump.

Reduced astrocytic coverage of synapses and the consequent increase in glutamate spillover could affect hippocampus-related behaviors in DTg mice. We detected no significant abnormalities in the open field, elevated plus maze, or home cage activity tests (Additional file 8: Figure S5), indicating that anxiety-related behaviors were not altered in the DTg mice. In the Morris water maze test, DTg mice had normal acquisition rates in the hidden platform task (Figure 5A), but demonstrated impaired spatial reference memory in the probe test (Figure 5B, *P* = 0.003, unpaired *t* test). DTg mice, however, spent significantly more time in the target quadrant (*F*_3,92_ = 27.006, *P* < 0.0001, non-repeated measures ANOVA), indicating that the memory impairment was not severe. Importantly, DTg mice treated with Dox (Figure 5, D and E) were not impaired in the probe test. The performance of the mice in the visible platform task did not differ across genotypes (Figure 5C). It should be noted that Dox treatment does not prevent leaky expression of Tg1 in a minor subset of dentate granule cells. These findings indicate that GST-IP_3_ sponge expression in the astrocytes of the DTg mice impairs spatial reference memory, which is closely linked to hippocampal function [25].

In the fear-conditioning test, DTg mice and WT mice exhibited an equivalent level of contextual and cued fear memory 1 and 2 d after training (Figure 5F). When assessed for remote fear memory 30 d post-training, however, DTg mice exhibited significantly reduced contextual fear memory (Figure 5G, *P* = 0.019, unpaired *t* test), whereas DTg and WT mice froze at similar levels when assessed for cued fear memory at 31 d (Figure 5G, *P* = 0.156, unpaired *t* test). DTg mice treated with Dox showed no significant defect in the recent or remote fear-conditioning tests (Figure 5, I and J). All mice tested showed comparable footshock sensitivity (Figure 5, H and K). These data suggest that GST-IP_3_ sponge expression in the astrocytes of the DTg mice impairs the establishment and/or retrieval of remote contextual fear memory, which is thought to involve interaction/communication between the hippocampus and neocortex after the formation of recent memory in the hippocampus [26-28].

## Discussion

The IP_3_ sponge, a novel recombinant hyperaffinity IP_3_ absorbent [13], inhibits IICR in cultured mammalian cells, starfish oocytes, and *Drosophila*[13,29,30]. In the present study, we generated DTg mice in which the GST-IP_3_ sponge was expressed in astrocytes using the Tet-OFF system [31]. This transgenic mouse system enabled us to induce the expression of the lacZ marker protein in approximately 90% of S100B-positive astrocytes in the hippocampal CA1 region of the DTg mice. Ca^2+^ imaging of hippocampal slices from transgenic mice expressing G-CaMP2 confirmed that agonist-evoked IICR was attenuated in the processes of GST-IP_3_ sponge-positive astrocytes. Thus, we obtained a mouse model in which astrocytic IICR was attenuated. Analyses of the DTg mice revealed 1) a significant reduction in the astrocyte coverage of asymmetric synapses in the hippocampal CA1 region, 2) enhanced glutamate spillover at CA1-CA3 synapses during high-frequency activity, and 3) impaired spatial reference memory and remote contextual fear memory.

IICR-dependent regulation of astrocytic ensheathment of synapses provides new insight into the controversy surrounding whether astrocytic Ca^2+^ signaling affects neuronal functions. These findings raise intriguing possibilities regarding neuron-astrocyte interactions: when the synapse releases glutamate on metabotropic glutamate receptors in the surrounding astrocyte, the level of Ca^2+^ activity in astrocytic processes is maintained, providing high astrocytic coverage of the synapse. An inactive synapse would then not have astrocytic coverage, allowing for neurotransmitter diffusion in and out of the synaptic cleft. Thus, our data provide a possible mechanism for activity-dependent regulation of astrocytic coverage in the tripartite synapse.

Indeed, astrocytic processes are considered very plastic [32-37], as is astrocytic coverage of synapses [38,39]. The dynamic motility of astrocytic processes is essential for astrocytes to modulate synaptic activity, but its regulation in vivo remains largely unknown. Glutamate causes filopodia formation in cultured hippocampal astrocytes [37], which is mediated by metabotropic glutamate receptors [40]. Metabotropic glutamate receptor-activation in astrocytes in vitro [41-44] and in situ [45] leads to an increase in intracellular Ca^2+^ concentrations through the release from intracellular stores. Our results provide a link between IICR and plasticity of the astrocytic processes in vivo. The downstream cascade leading to changes in the morphology of astrocytic processes, however, remains to be clarified, and might involve regulation of the organization and assembly of the actin cytoskeleton in astrocytes [40]. IICR in the growth cone acts on downstream effectors to regulate microtubule assembly and promote neurite extension [19]. Microtubule networks and actin networks might contribute to the motility of peripheral astrocytic processes in vivo. Moreover, the Ca^2+^ concentration regulates the migration of astrocytoma cells by forming or disassembling focal adhesions [44,45]. Local concentrations of Ca^2+^ are further suggested to be involved in filopodia formation by regulating focal adhesions [46]. These mechanisms might cooperatively contribute to the structural integrity of tripartite synapses.

Several reports suggest Ca^2+^-dependent release of glutamate and D-serine from astrocytes [6-8,47-49]. Both are co-agonists of NMDA receptors. Therefore, reduced Ca^2+^ activity in astrocytes might decrease the activation of NMDA receptors by those gliotransmitters during synaptic transmission. In contrast, we found that high-frequency stimulation in DTg mice increases the NMDA receptor current in CA1 pyramidal neurons, which correlates with enhanced glutamate spillover. Recent reports suggest that NMDA receptors located at synaptic and extrasynaptic sites have distinct functional roles [24,50]. Spillover can actually increase the proportion of extrasynaptic NMDA receptors activated during burst firing, affecting hippocampal-based learning. In addition, glutamate spillover leads to an increase in intersynaptic crosstalk. The increased intersynaptic crosstalk could impair the independent operation of hippocampal synapses and thus affect synaptic characteristics, such as the spatial precision of synaptic inputs [51], which might also be important for learning and memory. We cannot exclude the possibility that glutamate spillover during burst firing increases release probability by activating presynaptic NMDA receptors, which leaves open the possibility that reduced astrocytic coverage has presynaptic effects as well as postsynaptic effects. In the present study, however, it is unlikely that presynaptic mGluRs were involved in the enhanced NMDA receptor currents. NMDA receptor currents were recorded in the presence of S-MCPG (200 μM), which blocks Type I and Type II mGluRs. Activation of Type III mGluRs by enhanced glutamate spillover decreases the release probability at CA3-CA1 synapses with Shaffer collaterals [52]. If this were the case, the observed potentiation of the NMDA receptor current could be underestimated in GST-IP_3_ sponge-expressing animals. These and other effects of enhanced spillover on the hippocampal network should be addressed in future studies.

Concerning changes in long-term plasticity in DTg mice, we found that there were no significant differences in long-term potentiation (LTP), long-term depression (LTD), and depotentiation induced by conventional protocols in situ (i.e., 2 tetanic stimulations [5], 5 theta-burst stimulations (TBS) [53], and 3 TBS for LTP [53]; low-frequency stimulation (LFS) [54] and paired pulse-LFS [55] for LTD; and 2 tetanic stimulations followed by LFS for depotentiation [54]; data not shown). Despite these findings, our results indicate that enhanced glutamate spillover in the hippocampus correlates with impaired learning and memory in DTg mice, very likely by perturbing the independent operation of hippocampal synapses [51]. Indeed, if LTP can be effectively triggered, but in the ‘wrong’ population of synapses, the memory still can be potentially affected.

The normal recent context fear memory in DTg mice contrasts with the impaired spatial reference memory in these mice. This discrepancy can be reconciled by the fact that spatial reference memory evaluated in the water maze is not associated with contextual fear memory [56-58] and each of these distinct types of memories have different underlying mechanisms [59]. Although the impairment of remote contextual fear memory in DTg mice indicates miscommunication between the hippocampus and cortical areas [26-28], our result does not exclude the possibility that the anterior cingulate cortex, in which GST-IP_3_ sponge expression in the astrocytes was also observed (Figure 1C and data not shown), is solely responsible for the phenotype [27,60,61].

## Conclusions

**Figure 6:**
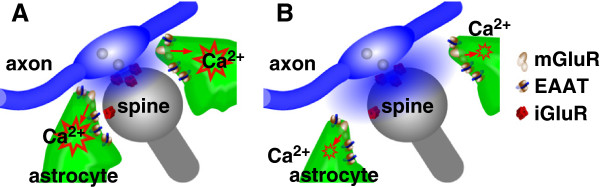
**Involvement of astrocytic Ca**^**2+**^**in tripartite synapses.** (**A**) Control astrocytes. (**B**) IP_3_ sponge-expressing astrocytes. Suppression of Ca^2+^ activity in IP_3_ sponge-expressing astrocytes leads to retraction of astrocytic processes from the synapse, which moves astrocytic glutamate transporters further away and allows for more glutamate spillover.

Our findings provide evidence that IP_3_-mediated astrocytic Ca^2+^ activity regulates synaptic coverage by astrocytes in the hippocampal CA1 (Figure 6), which confers normal glutamate clearance to improve input specificity and affects hippocampus-dependent learning and memory.

## Methods

### Generation of *GST-IP*_*3*_*sponge-TetO-nls-LacZ* (*Tg1*) and *GLT-1-tTA* (*Tg2*) transgenic mice

All experimental protocols were approved by the RIKEN Institutional Animal Care and Use Committee. For the *Tg1* DNA construct, coding sequences of the GST-IP_3_ sponge [13] and nls-LacZ [62], together with splicing signals derived from the pMSG vector (GE Healthcare), were inserted downstream of the bidirectional tetracycline-Operator (TetO) element-dependent promoters of the pBI Tet vector (Clontech). For the *Tg2* DNA construct, the tTA coding sequence from the pTet-Off vector (Clontech), with splicing signals derived from the pMSG vector (GE Healthcare), was inserted using a *Red/ET* Recombination system (Gene Bridges) into a bacterial artificial chromosome clone, RP23-361H22 (BAC PAC Resources), containing the *GLT-1* (*Slc1a2*) gene to replace the first ATG of the *GLT-1* coding region. These constructs were digested away from the prokaryotic sequence, purified, and individually injected into the pronuclei of C57BL/6-fertilized embryos to generate transgenic offspring. Each founder line was backcrossed with C57BL/6 mice and maintained in this genetic background.

### Astrocyte Ca^2+^ imaging with G-CaMP2

#### Slice preparation

Transverse hippocampal slices were prepared from 5- to 6-mo-old Ctrl mice carrying *tetO-G-CaMP2*[14] and *GLT-1-tTA* (*Tg2*), and TTg mice carrying *GST-IP*_*3*_*sponge-TetO-nls-LacZ* (*Tg1*), *tetO-G-CaMP2* and *GLT-1-tTA* (*Tg2*) in accordance with RIKEN regulations. Animals were anesthetized with 2-bromo-2-chloro-1,1,1-trifluroethane and decapitated. The brain was exposed, chilled with ice-cold solution containing (in mM): 75 sucrose, 87 NaCl, 2.5 KCl, 0.5 CaCl_2_, 1.25 NaH_2_PO_4_, 7 MgCl_2_, 25 NaHCO_3_, 1 Na-ascorbate, and 11 D-glucose. Hippocampi from both hemispheres were isolated and placed in an agar block. Transverse slices (350-400 μm) were cut with a vibrating microtome (Microm HM 650V, Thermo Fisher Scientific Inc.) and left to recover for 30 min at 34°C and then at room temperature for 1 h in an interface chamber with storage solution containing (in mM): 127 NaCl, 2.5 KCl, 1.25 NaH_2_PO_4_, 1 MgCl_2_, 1 CaCl_2_, 25 NaHCO_3_, and 25 D-glucose. The slices were then transferred to the recording chamber and continuously perfused with recording solution containing (in mM): 127 NaCl, 2.5 KCl, 1.25 NaH_2_PO_4_, 1 MgCl_2_, 2 CaCl_2_, 25 NaHCO_3_, and 25 D-glucose. All solutions were saturated with 95% O_2_ and 5% CO_2_. Osmolarity was adjusted to 300 ± 5 mOsm.

#### Two-photon imaging

Hippocampal slices were transferred to the recording chamber mounted on the stage of the Olympus BX-61 equipped with differential interference contrast optics and a water immersion objective lens (60x, NA = 0.9, Olympus) superfused with artificial cerebrospinal fluid (ACSF). A mode-locked tunable 720–930 nm laser Chameleon XR (Coherent) was used as the excitation source. Fluorescence was collected using photomultiplier tubes (Hamamatsu). Serial scanning of slices was tuned to an 890-nm wavelength at 1 Hz and emitted green fluorescence was collected through a 495–540 nm bandpass filter. Astrocytes were selected for data analysis if they emitted G-CaMP2 fluorescence, and spontaneous Ca^2+^ transients were recorded during the first 5 min of each recording. Cells with frequent (> 0.0167 Hz) somatic Ca^2+^ activity were excluded. Stimulation-induced Ca^2+^ transients were initiated by pressure-ejection of 200 μM DHPG in ACSF containing 50 μM Alexa594 (Molecular Probes) through a glass micropipette (2–3 μm diameter). The puff pipette was always kept 30 μm away from and 100 μm above the recorded astrocyte. Two 5-min long recordings were made for a long puff time (500 ms) and a short puff time (100 ms) in a random sequence. DHPG was applied four times at 1-min intervals at 1 min after the beginning of each 5-min long recording period.

#### Post-calcium imaging immunohistochemistry and confocal imaging

To confirm the expression of G-CaMP2 and the GST-IP_3_ sponge in the recorded astrocytes, slices from TTg mice were fixed with 4% paraformaldehyde immediately after recording and then processed for immunostaining using the anti-GFP antibody (for G-CaMP2) and anti-β-galactosidase as described in the Additional Methods (Additional file 4). Confocal images were acquired with an Olympus FV1000 confocal microscope. Astrocytes that showed double immunolabeling were recorded through 10x and 20x objectives and a single scan was performed by alternating the excitation wavelengths between 543 nm (G-CaMP2) and 488 nm (lacZ). Acquired images were analyzed using FluoView (Olympus) and ImageJ (a public domain Java image processing program by Wayne Rasband).

#### Data analysis for two-photon Ca^2+^ imaging

Two-photon Ca^2+^ imaging data was analyzed using a custom-written algorithm in MATLAB (MathWorks). Statistical analysis was performed with SPSS (IBM). Time series of fluorescence changes were standardized along the time axis pixel-by-pixel with subtraction of the mean and division of the SD. The images at each time were then flattened by Gaussian filter (4 pixels, σ = 2.0) to reduce the shot noise of the imaging device. We calculated the SD of the filtered image stacks along the time axis pixel-by-pixel and detected changeable areas that exceeded a criterion (from mean + 10SD to + 12SD). Those areas were denoised and smoothed by morphologic operations, and then identified as regions of interest (ROIs). The soma area was distinguished from process regions that we used as ROIs. The standardized time series were rescaled to original fluorescence changes pixel-by-pixel with multiplication of the original SD and addition of the original mean. We created time traces by pixel-averaging within each ROI. The mean and SD of time points less than median + 1SD was defined as the baseline and base SD of the time trace, respectively. Time traces were smoothed by low-pass filter (cutoff frequency, 0.2 Hz). Ca^2+^ events were identified as follows: We set two criteria to identify Ca^2+^ events: baseline + 1-base SD line (first criterion) and baseline + 2-base SD line (second criterion). Traces with peaks larger than the second criterion were considered candidate events. If the trace fell below the first criterion before and after the peak, the first points were defined as the start and end points of one event, respectively. Time duration from the start to the end point was defined as one event duration. The fractional change in fluorescence intensity was defined as *ΔF*/*F*_0_ = (*F* – *F*_0_) / *F*_0_, where *F*_0_ was the baseline and *F* is the time trace. Event areas were computed with summation of *ΔF*/*F*_0_ during the event duration. Durations and event areas of individual Ca^2+^ events were averaged over events, trials, and samples under each puff condition. The durations and event areas were tested using two-way ANOVA and multiple comparisons with Bonferroni’s correction.

### Electron microscopy

#### Sample preparation

Mice were deeply anesthetized by intraperitoneal injection of sodium pentobarbital (0.1 mg/g body weight). After transcardial perfusion with 10 ml saline solution, the brains were fixed by perfusion with 100 ml 2% paraformaldehyde and 2.5% glutaraldehyde in 0.1 M cacodylate buffer (pH 7.3) at 4°C. Brain tissues were dissected into small blocks (~3-mm thick) and postfixed in the same fixative overnight at 4°C. Tissue blocks were coronally sliced (200-μm thick) on a DTK-100 microslicer (Dosaka) and postfixed with 1% osmium tetroxide in 0.1 M cacodylate buffer at 4°C for 1 h. Tissue slices were stained with 1% uranyl acetate at room temperature for 1 h and then dehydrated, infiltrated, and embedded in plastic resin. Semi-thin (590-nm thick) sections were cut with an Ultratrim Diatome (Nisshin EM) on an Ultracut E Ultramicrotome (Reichert-Jung) and stained with 0.25% toluidine blue. Stained sections were viewed with a BX50 microscope (Olympus) and corresponding small areas (~0.5 x 1.0 mm) of the hippocampal CA1 region in each sample were trimmed. Ultrathin (90-nm-thick) sections were cut with an Ultra 45° Diatome (Nisshin EM) on an ultramicrotome, stained with 4% uranyl acetate and then with 0.4% lead citrate, and subjected to electron microscopy analysis in a JEM 1010 (JEOL Company) at an acceleration voltage of 80 kV. Electron micrographs were obtained from the middle one-third of the CA1 *stratum radiatum* of the dorsal hippocampus (approximately coronal section 300) [63]*.* Electron microscopy pictures at a magnification of 15,000x were scanned at a resolution of 720 dpi with an ES-2200 image scanner (Epson) to acquire the digital image and synaptic morphology was analyzed.

#### Astrocytic coverage of synapses and synaptic density

Asymmetric synapses, representing excitatory terminals of Schaffer collaterals, were evaluated in the *stratum radiatum* subfield of the CA1 region. Astrocytic coverage of synapses was classified into two types: synapses without astrocytic contact and synapses with astrocytic contact. Percentages of each type of synapse and the synaptic density (number of synapses/μm^2^ neuropil) were assessed using 20 electron micrographs from two mice of each genotype (5-mo-old for Figure 3C and 11-mo-old for Figure 3D), two sample blocks per animal, and five randomly selected pictures per sample block.

### Recording of STCs in hippocampal astrocytes

#### Slice preparation

Hippocampal slices were prepared from 4- to 5-week-old WT and DTg mice as described above.

#### Whole-cell recording from astrocytes

Whole-cell recordings were made with a Multiclamp 700B amplifier (Axon Instruments). The patch pipettes (3–7 MΩ) were filled with solution containing (in mM) 135 KCH_3_SO_3_, 10 HEPES, 10 Na-phosphocreatine, 4 MgCl_2_, 4 Na-ATP, and 0.4 Na-GTP; 290 mOsm, pH 7.2. Picrotoxin (100 μM), 2,3-dioxo-6-nitro-1,2,3,4-tetrahydrobenzo[f]quinoxaline-7-sulfonamide (NBQX, 25 μM), DL-2-amino-5-phosphonopentanoic acid (50 μM), and 8-cyclopentyltheophylline (4 μM) were added to recording ACSF to block GABA_A_, AMPA/kainate, NMDA, and adenosine receptors, respectively. Astrocytes in the CA1 *stratum radiatum* were identified by their small cell bodies, low input resistance, and very negative resting membrane potential [64]. The cells were voltage-clamped at their resting membrane potential. The current recordings were filtered at 2 kHz, digitized at 10 kHz with a PCI-6221 A/D board (National Instruments), and recorded with WinWCP software (Strathclyde University, Glasgow, UK). STCs were evoked with a bipolar stimulating electrode placed in the *stratum radiatum*. Access resistance was monitored throughout each experiment. Experiments were excluded from the data analysis when the access resistance changed by more than 20%.

#### Analysis

The first response to paired stimulation was scaled to the response elicited by a single stimulus, and then the response to the single stimulus was subtracted from the scaled response to the paired stimulation. The response to the single stimulus was then subtracted from the pure second response. The difference represented the STC without the potassium current [22]. The STC was used to estimate the rise and decay time of the baseline transporter current.

In an experiment with a series of stimuli, three different stimulation patterns were used: a single stimulus, 4 stimuli, and 5 stimuli. To compare the kinetics of the response to the 1^st^ and 5^th^ stimuli, the response to 4 stimuli was subtracted from the response to 5 stimuli. The pure 5^th^ response was used for further analysis.

### Neuronal NMDA current

#### Slice preparation

Hippocampal slices were prepared from 4- to 5-week-old WT and DTg mice as described above.

#### Whole-cell recording from CA1 pyramidal neurons

Whole-cell patch-clamp recordings of CA1 pyramidal neurons were made in ACSF containing picrotoxin (100 μM), CGP52432 (5 μM), S-MCPG (200 μM), and NBQX (25 μM) to isolate NMDA EPSCs. For this experiment, the pipette solution contained (in mM): 130 CsCH_3_SO_3_, 8 CsCl, 10 HEPES, 4 Mg-ATP, 0.4 Na_2_-GTP, 10 Na_2_-phosphocreatine, and 5 QX-314, pH 7.2; osmolarity was 290 mOsm. To evoke NMDA EPSCs, one bipolar stimulating electrode was positioned on the Schaffer collateral pathway, and a stimulus was generated every 30 s by a constant-current stimulator (Digitimer). Experiments were always started 15 min after breaking the seal to provide for sufficient diffusion of the intracellular channel inhibitors. Whole-cell NMDA currents in response to Schaffer collateral stimulation were measured in voltage-clamp mode at +40 mV. Series resistance was monitored before each step by measuring the peak current in response to a −3 mV voltage step. Cells with unstable series resistance (> 20% change) were excluded from further analysis.

### Behavioral analysis

All experiments were performed by an investigator blind to the mouse genotype.

#### Morris water maze test

The experiments were conducted with 5-mo-old male mice. The Morris water maze test was performed as previously described with some modifications [33]. Mice were given 4 trials per day for 7 consecutive days in the hidden platform task. A randomly selected starting point along the rim of the maze was used for each of the four trials. A probe test was performed on day 8 after the acquisition session. In the probe test, the platform was removed from the tank and each mouse was allowed to swim for 60 s. On day 10, mice were tested in a visible platform task for 3 consecutive days. In the visible platform task, the platform was made visible by attaching a black cubic landmark to the platform. Mouse movement in the water maze was recorded by a video camera and analyzed using NIH image WM software (O’Hara &Co.).

#### Contextual and cued fear-conditioning test

The experiments were conducted with 5.5-mo-old male mice. A fear-conditioning shock chamber (10 x 10 x 10 cm) was used. Each mouse was placed into the conditioning chamber and allowed to explore for 2 min. The mouse then received two tone-shock pairings with a 60-s interstimulus interval. Each tone-shock pairing comprised an auditory cue (80 dB white noise, 30 s long) that co-terminated with an electric footshock (0.75 mA, 2 s long). The mice were left in the conditioning chamber after the last tone-shock pairing for 1 min. The 24-h contextual test was performed 24 h after the conditioning session. Mice were returned to the same conditioning chamber and their behavior was monitored for 5 min without tone or shock. The cued test was performed 24 h after the contextual test. Another testing chamber with different properties (shape, floor type, and wall color of the surrounding environment) was used. The mice were placed in the chamber and allowed to explore for 2 min. The same tone used in the conditioning session was given for 3 min. The remote contextual fear test was repeated 30 d after the conditioning session, and the remote cued test was also repeated 24 h later. The freezing behavior of the mice was monitored using a video camera and images were processed with NIH Image FZ software (O’Hara &Co.).

#### Footshock sensitivity test

After the remote fear memory test, footshock sensitivity was examined by giving mice electrical shocks of increasing intensity, ranging from 0.05 mA to 1 mA, and monitoring their behavior (flinching, vocalization, small jump, and big jump).

## Competing interests

The authors declare that they have no competing interests.

## Authors’ contributions

MT, PYS, AS, and SI conceived the study. MT, PYS, HG, TY, AS, and SI wrote the paper. MT, PYS, HG, JN, and RA performed the experiments. TY analyzed the calcium imaging data. TF and KM contributed to creation and characterization of GST-IP_3_ sponge. All authors have read and approved the manuscript.

## Supplementary Material

Additional file 1 Table S1 Cell type of lacZ-positive cells in specific brain areas of DTg mice. The NeuN-positive lacZ cells in the DG appear to correspond to the leakage observed in *Tg1* mice (Figure 1D). Click here for file

Additional file 2 Figure S1 Cell count of S100B-positive cells. Frozen brain sections (20 μm, n = 9–12) of 4-mo-old WT and DTg mice (N = 2 for each genotype) were labeled with anti-S100B antibody. Immunofluorescent images using an objective lens (20×) were acquired with a CCD camera and the number of S100B-positive cells was counted in the somatosensory cortex (SC), hippocampal CA1 (CA1), dentate gyrus (DG), and amygdala (Am). Results are provided as mean ± SEM. Click here for file

Additional file 3 Figure S2 Detection and quantification of GST-IP_3_ sponge expression. (A) The GST-IP_3_ sponge was detected in DTg mouse brains using a glutathione affinity trap. (B) Estimation of the concentration of the extracted recombinant GST-IP_3_ sponge (G224/R441Q). Serially diluted bovine serum albumin and recombinant protein in polyacrylamide gels were stained with Coomassie Brilliant Blue and quantified by densitometric scanning. The concentration of the original elution of recombinant protein was estimated to be 0.88 mg/ml using a standard curve of bovine serum albumin. Arrowhead indicates the molecular size of the recombinant protein (66 kD). (C – E) Quantification of the GST-IP_3_ sponge by glutathione-trapping. (C) Expression of the GST-IP_3_ sponge in the brains of WT, *Tg1*, and DTg mice at 3 mo of age supplied with (On) or without (Off) Dox in the drinking water and beginning the supply of Dox water at 1 mo after birth (Off/On). (D) Changes in the GST-IP_3_ sponge expression over time in DTg mice supplied with normal water (Off) at 1, 3, 5, and 9 mo of age and with Dox water from 1 mo after birth (Off/On) to 3, 5, and 9 mo of age. (E) Quantification of the GST-IP_3_ sponge analyzed (D) using standard curves obtained from band densities of glutathione-trapping of diluted recombinant proteins (range 9.2 - 293.2 pg). Results are provided as mean ± SEM [N = 3, except for DTg mice supplied with normal water at 1 mo of age (N = 1) ]. Dox* indicates Dox treatment starting at 1 mo of age. Click here for file

Additional file 4 Additional Methods Mouse breeding, Doxycycline (Dox) treatment, LacZ or hematoxylin staining of cryosections, Immunofluorescent microscopy, Counting lacZ-positive cells co-stained with marker protein, Detection of GST-IP_3_ sponge by glutathione-trapping, Quantification of GST-IP_3_ sponge expression, Electrophysiology, Behavioral analysis. Click here for file

Additional file 5 Table S2 Number of Ca^2+^ events shown in Figure 2O and P. Click here for file

Additional file 6 Figure S3 Double immunofluorescence analysis of hippocampal CA1 of WT and DTg mice using antibodies against GLT-1 and S100B. Sections were treated with anti-GLT-1 (A, D) or anti-S100B (B, E) antibodies in WT (A – C) and DTg (D – F) mice. Expression levels of GLT-1 and S100B in DTg mice (D, E) are comparable to those in WT mice (A, B). Scale bar, 50 μm. Click here for file

Additional file 7 Figure S4 Normal electrophysiologic properties of the DTg mouse hippocampus. Hippocampal slices obtained from DTg mice showed a normal input–output relationship (A) and paired pulse facilitation (PPF) (B). Insets represent sample traces. fEPSP, extracellular field excitatory postsynaptic potentials. PSFV, presynaptic fiber volley. ISI, interstimulus intervals. Sample numbers (“n” indicates number of slices; “N” represents number of animals.); Input–output curves, n = 15, N = 8 for WT, n = 14, N = 7 for DTg; PPF, n = 13, N = 8 for WT, n = 13, N = 7 for DTg. The values on the graphs represent mean ± SEM. Click here for file

Additional file 8 Figure S5 Unaltered anxiety-related behaviors in DTg mice. (A – C) Performance of DTg mice (N = 12) and their WT littermates (N = 12) in an open field test. The difference in total distance traveled (*P* = 0.95) or time spent in the center (*P* = 0.19) were not significantly different between genotypes. (D) Performance in the elevated plus maze task of DTg mice (N = 12) and their WT littermates (N = 12). Time spent in the open arms (*P* = 0.17), closed arms (*P* = 0.16), or center (*P* = 0.35) were not significantly different between genotypes. (E) Home cage activity on day 6 was not significantly different in daytime (*P* = 0.63) or nighttime (*P* = 0.92) activity between genotypes (N = 12 for each genotype). Unpaired *t* test was used for statistical analysis. Data represent mean ± SEM. Click here for file
