# Supplementary figures and images for: Astrocytic Ca2+ signals are required for the functional integrity of tripartite synapses
Source: Mol Brain. 2013 Jan 28;6:6. doi: 10.1186/1756-6606-6-6 (PMC3563617; doi:10.1186/1756-6606-6-6)

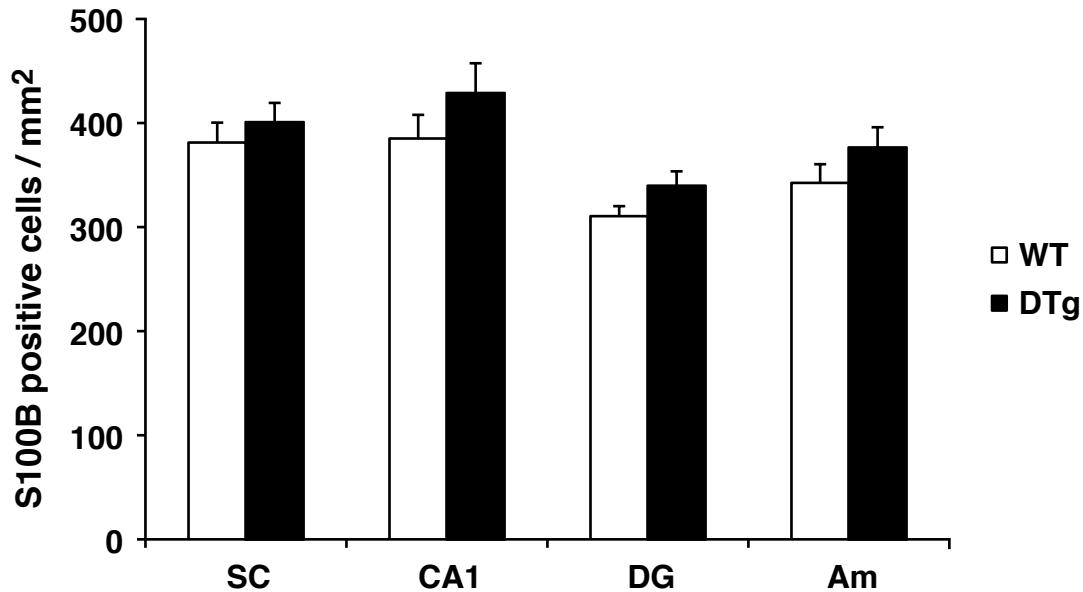

Supplement: Additional file 2 Figure S1 — Cell count of S100B-positive cells. Frozen brain sections (20 μm, n = 9–12) of 4-mo-old WT and DTg mice (N = 2 for each genotype) were labeled with anti-S100B antibody. Immunofluorescent images using an objective lens (20×) were acquired with a CCD camera and the number of S100B-positive cells was counted in the somatosensory cortex (SC), hippocampal CA1 (CA1), dentate gyrus (DG), and amygdala (Am). Results are provided as mean ± SEM. [file 1756-6606-6-6-S2.pdf]

**A**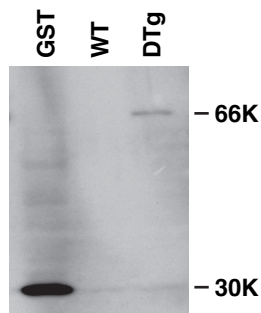**B**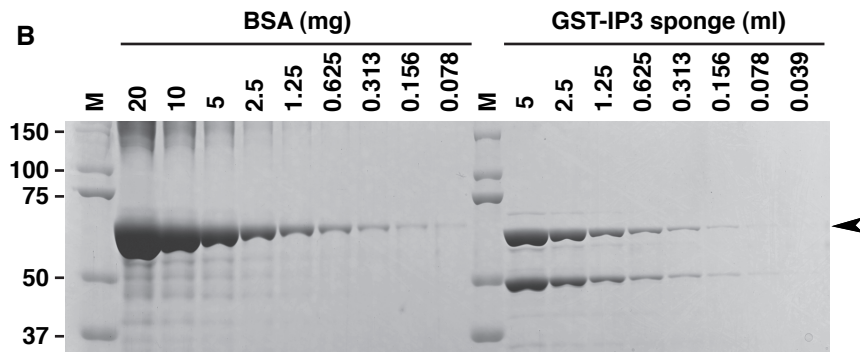**C**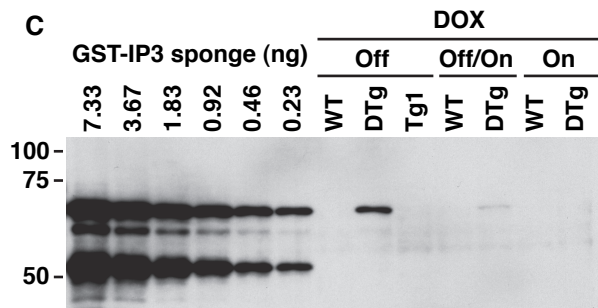**D**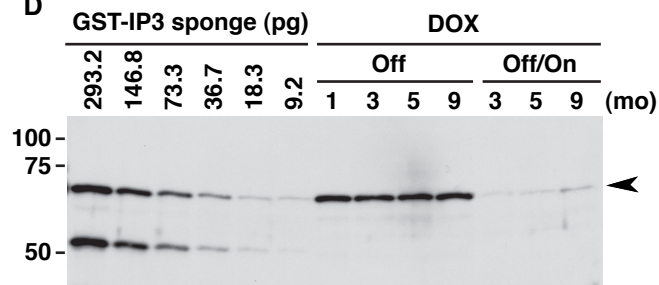**E**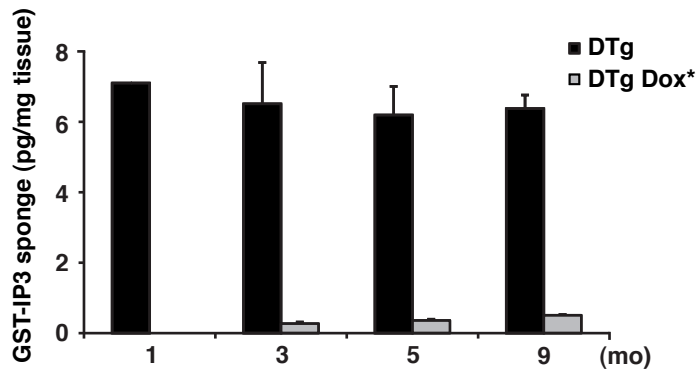

Supplement: Additional file 3 Figure S2 — Detection and quantification of GST-IP3 sponge expression. (A) The GST-IP3 sponge was detected in DTg mouse brains using a glutathione affinity trap. (B) Estimation of the concentration of the extracted recombinant GST-IP3 sponge (G224/R441Q). Serially diluted bovine serum albumin and recombinant protein in polyacrylamide gels were stained with Coomassie Brilliant Blue and quantified by densitometric scanning. The concentration of the original elution of recombinant protein was estimated to be 0.88 mg/ml using a standard curve of bovine serum albumin. Arrowhead indicates the molecular size of the recombinant protein (66 kD). (C – E) Quantification of the GST-IP3 sponge by glutathione-trapping. (C) Expression of the GST-IP3 sponge in the brains of WT, Tg1, and DTg mice at 3 mo of age supplied with (On) or without (Off) Dox in the drinking water and beginning the supply of Dox water at 1 mo after birth (Off/On). (D) Changes in the GST-IP3 sponge expression over time in DTg mice supplied with normal water (Off) at 1, 3, 5, and 9 mo of age and with Dox water from 1 mo after birth (Off/On) to 3, 5, and 9 mo of age. (E) Quantification of the GST-IP3 sponge analyzed (D) using standard curves obtained from band densities of glutathione-trapping of diluted recombinant proteins (range 9.2 - 293.2 pg). Results are provided as mean ± SEM [N = 3, except for DTg mice supplied with normal water at 1 mo of age (N = 1) ]. Dox* indicates Dox treatment starting at 1 mo of age. [file 1756-6606-6-6-S3.pdf]

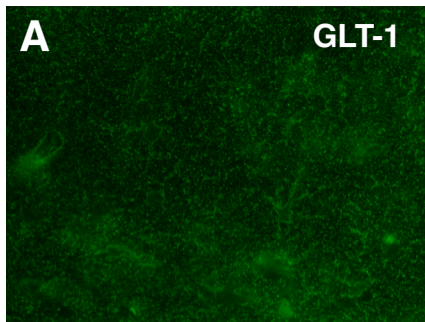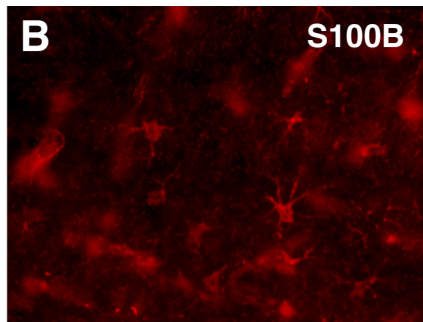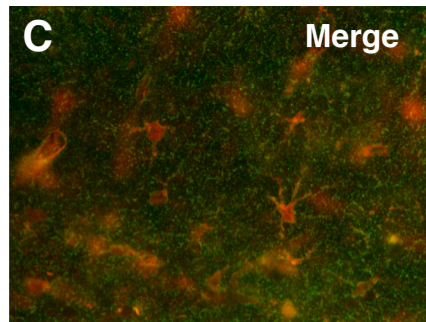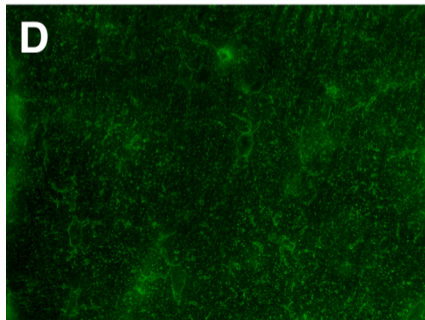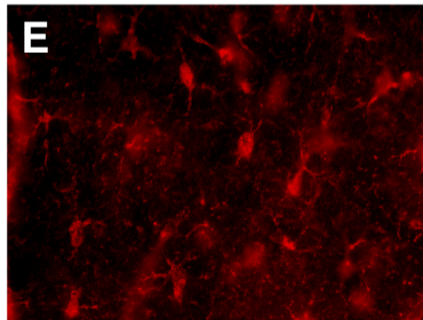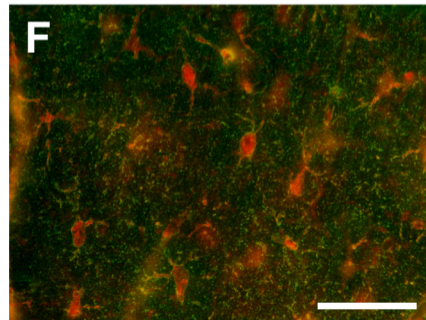

Supplement: Additional file 6 Figure S3 — Double immunofluorescence analysis of hippocampal CA1 of WT and DTg mice using antibodies against GLT-1 and S100B. Sections were treated with anti-GLT-1 (A, D) or anti-S100B (B, E) antibodies in WT (A – C) and DTg (D – F) mice. Expression levels of GLT-1 and S100B in DTg mice (D, E) are comparable to those in WT mice (A, B). Scale bar, 50 μm. [file 1756-6606-6-6-S6.pdf]

**A**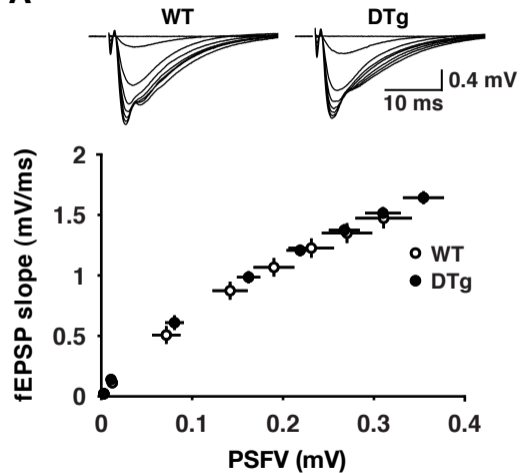**B**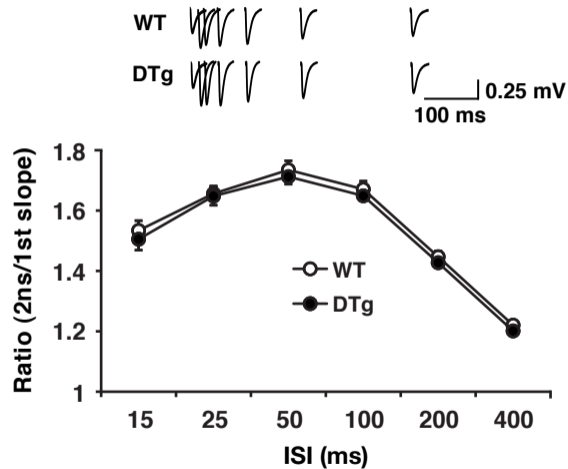

Supplement: Additional file 7 Figure S4 — Normal electrophysiologic properties of the DTg mouse hippocampus. Hippocampal slices obtained from DTg mice showed a normal input–output relationship (A) and paired pulse facilitation (PPF) (B). Insets represent sample traces. fEPSP, extracellular field excitatory postsynaptic potentials. PSFV, presynaptic fiber volley. ISI, interstimulus intervals. Sample numbers (“n” indicates number of slices; “N” represents number of animals.); Input–output curves, n = 15, N = 8 for WT, n = 14, N = 7 for DTg; PPF, n = 13, N = 8 for WT, n = 13, N = 7 for DTg. The values on the graphs represent mean ± SEM. [file 1756-6606-6-6-S7.pdf]

**A**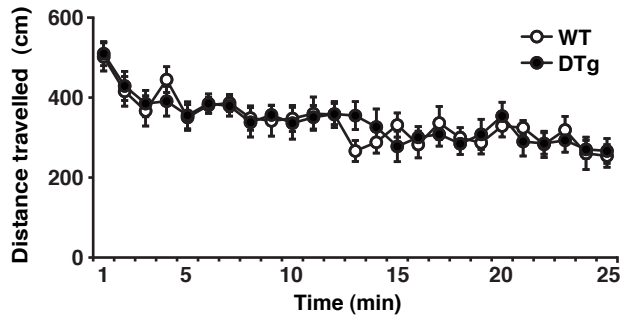**B**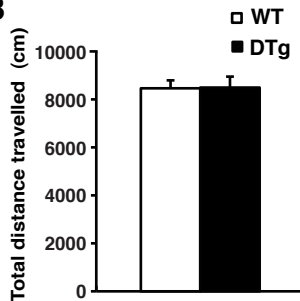**C**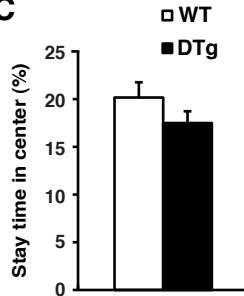**D**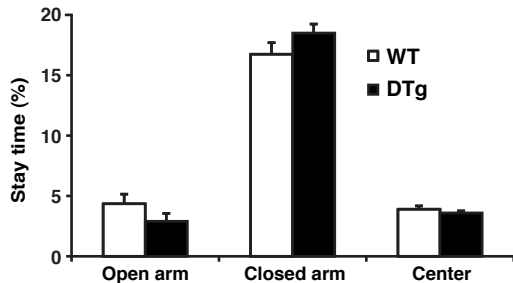**E**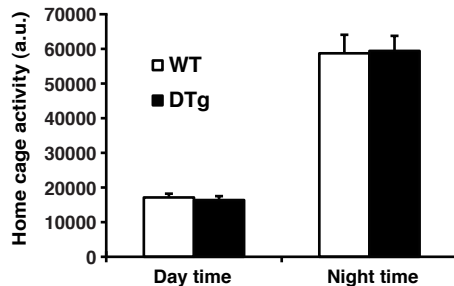

Supplement: Additional file 8 Figure S5 — Unaltered anxiety-related behaviors in DTg mice. (A – C) Performance of DTg mice (N = 12) and their WT littermates (N = 12) in an open field test. The difference in total distance traveled (P = 0.95) or time spent in the center (P = 0.19) were not significantly different between genotypes. (D) Performance in the elevated plus maze task of DTg mice (N = 12) and their WT littermates (N = 12). Time spent in the open arms (P = 0.17), closed arms (P = 0.16), or center (P = 0.35) were not significantly different between genotypes. (E) Home cage activity on day 6 was not significantly different in daytime (P = 0.63) or nighttime (P = 0.92) activity between genotypes (N = 12 for each genotype). Unpaired t test was used for statistical analysis. Data represent mean ± SEM. [file 1756-6606-6-6-S8.pdf]
