# Supplementary material for: Astrocytic Ca2+ signals are required for the functional integrity of tripartite synapses
Source: Mol Brain. 2013 Jan 28;6:6. doi: 10.1186/1756-6606-6-6 (PMC3563617; doi:10.1186/1756-6606-6-6)
Supplement: Additional file 4 Additional Methods — Mouse breeding, Doxycycline (Dox) treatment, LacZ or hematoxylin staining of cryosections, Immunofluorescent microscopy, Counting lacZ-positive cells co-stained with marker protein, Detection of GST-IP3 sponge by glutathione-trapping, Quantification of GST-IP3 sponge expression, Electrophysiology, Behavioral analysis. [file 1756-6606-6-6-S4.docx]

**Additional Methods.**

**Mouse breeding**

After characterizing several founder mice by crossing them with the *CamKIIa-tTA* Tg line [1] and lacZ staining, the *Tg1* line with the strongest lacZ staining with *CamKIIa-tTA* Tg, but the lowest leakage without *CamKIIa-tTA* Tg, was selected. The selected *Tg1* line was then crossed with *Tg2* lines to generate double transgenic (DTg) mice heterozygous for both transgenes. We selected one of the *Tg2* lines with astrocyte-specific gene induction throughout the brain except the cerebellum to avoid potential motor deficits. The selected *Tg1* and *Tg2* lines were crossed to obtain DTg and wild-type (WT) mice used for further studies. Tail DNA from all offspring was genotyped by polymerase chain reaction (PCR) to detect the presence of each transgene separately. For *Tg1*, PCR primers 5'-GTGATGCTGAAAATTGGTAC-3' and 5'-GCTTCGTGACAGACCTTCTC-3' were used to detect the 186-bp sequence present in the IP_3_-sponge. For *Tg2*, PCR primers 5'-ATTACGGGTCTACCATCGAGG-3' and 5'-GTCCAGATCGAAATCGTCTAG-3' were used to detect the 221-bp sequence present in tTA.

**Doxycycline (Dox) treatment**

Some mice were reared in the presence of Dox (25 µg/ml in the drinking water) with administration initiated before birth (Dox), and some mice were treated with Dox beginning at 1 mo of age (Dox*).

**Whole-mount β-galactosidase (lacZ) staining of the brain**

Whole-mount lacZ staining was performed as described previously [2]. One-month-old mice were deeply anesthetized with halothane and subjected to cervical dislocation. Their brains were excised immediately, cut parasagittally with a scalpel blade, and fixed in 0.2% glutaraldehyde, 2 mM MgCl_2_, and 5 mM EGTA in phosphate-buffered saline (PBS) on ice for 20 min. The brains were then washed in PBS on ice and stained in X-gal solution [1 mg/ml X-gal, 5 mM K_3_Fe(CN)_6_, 5 mM K_4_Fe(CN)_6_, 0.02% NP-40, 0.01% deoxycholate, and 2 mM MgCl_2_ in PBS] at 37°C overnight. LacZ-stained brains were then washed in PBS and maintained at 4°C prior to photographing.

**LacZ or hematoxylin staining of cryosections**

Four-month-old mice were deeply anesthetized with tribromoethanol (Avertin^®^) and fixed with 4% paraformaldehyde in PBS for 10 min via transcardial perfusion at 4°C. The brains were excised and postfixed with the same fixative for 2 h at 4°C. They were then washed in PBS and processed through a graded series of sucrose concentrations ranging from 15% to 30% in PBS at 4°C for 5 to 12 h for each concentration, bisected coronally with a scalpel blade, and frozen on dry ice. Frozen sections were cut at 50 μm using a Retoratome freezing microtome (YAMATO). For lacZ staining, the sections were stained in X-gal solution at 37°C overnight, washed with PBS, and mounted on slides. For hematoxylin staining, sections were stained with Hematoxylin 3G (Sakura Finetechnical).

**Immunofluorescent microscopy**

The mice were deeply anesthetized by intraperitoneal injection of tribromoethanol. The mice were first transcardially perfused with 10 ml saline at room temperature and then with 50 ml of 4% paraformaldehyde in 0.1 M sodium phosphate buffer (pH 7.3) at 4°C. The brains were removed, dissected into small coronal blocks (~5-mm thick), and postfixed with the same fixative overnight at 4°C. Brain tissues were equilibrated in 30% sucrose in 0.1 M phosphate buffer (pH 7.3) at 4°C and then frozen in Tissue-Tek OCT compound (Sakura Finetechnical). For immunohistochemistry, 20-µm thick coronal sections were prepared using a cryostat. Free-floating sections were washed with PBS (137 mM NaCl, 2.68 mM KCl, 8.10 mM Na_2_HPO_4_, 1.47 mM KH_2_PO_4_, pH 7.3) and permeabilized with PBS containing 0.1% Triton X-100 for 15 min. Nonspecific reactions were blocked by incubation with 5% normal goat serum and 50 mM NH_4_Cl in PBS for 30 min at room temperature. The sections were then incubated overnight at 4°C with the following primary antibodies; rabbit polyclonal antibodies specific for β-galactosidase (1:5000 or 1:10000; ICN Pharmaceuticals) and glutathione-S-transferase (GST; 1:100; MBL); mouse monoclonal antibodies specific for S100B (1:500; Sigma-Aldrich), NeuN (1:200; Chemicon International), and β-galactosidase (1:1000; Promega); rat monoclonal antibody specific for green fluorescent protein (to detect G-CaMP2; 1:250; Nacalei Tesque); guinea pig polyclonal antibody against glial glutamate transporter-1 (GLT-1; 1:500; Millipore). After repeated washing in PBS containing 0.025% Triton X-100 (PBS-T), the sections were incubated for 2 h at room temperature with the following secondary antibodies; goat Alexa 488-conjugated anti-rabbit IgG or goat Alexa 555/488-conjugated anti-mouse IgG or goat Alexa 546-conjugated anti-rat IgG or goat Alexa 488-conjugated anti-guinea pig IgG (1:2000; Molecular Probes/Invitrogen). After repeated washing in PBS-T, the sections were mounted on slide glass with 0.25% p-phenylenediamine dihydrochloride in 75% glycerol. Digitized images of immunofluorescent staining were acquired with a BX50 microscope equipped with an epifluorescence attachment and a DP-70 CCD (charge-coupled device) camera (Olympus) using an UplanApo objective lens (20x; Olympus). Four-month-old mice were used in Fig. 1, F and G and Fig. S3, and 1-mo-old mice were used in Fig. 1, H to J.

**Counting lacZ-positive cells co-stained with marker protein**

Double immunofluorescent labeling with anti-β-galactosidase and anti-S100B antibodies, or with anti-β-galactosidase and anti-NeuN antibodies was used to identify cells expressing lacZ and specific marker proteins for astrocyte and neurons in DTg mice (N = 2, 4 mo old). The numbers of cells that were lacZ-positive/S100B-positive, lacZ-positive/NeuN-positive, and S100B-positive/lacZ-positive were counted in the somatosensory cortex, hippocampal CA1, dentate gyrus, and amygdala.

**Detection of GST-IP_3_ sponge by glutathione-trapping**

The GST-IP_3_-sponge was detected by glutathione-trapping. For glutathione-trapping, a frozen (-80°C) stock of brain hemispheres was weighed and homogenized in 5 ml of ice-cold homogenate buffer containing 50 mM Tris-HCl (pH 8.0), 50 mM NaCl, 1 mM EDTA, 1 mM dithiothreitol (DTT), and protease inhibitor cocktail (Complete Mini, Roche Diagnostics GmbH) using a Teflon-glass homogenizer at a pestle speed of 3300 rpm and 20 strokes. The homogenate was centrifuged at 9400x g for 20 min at 4°C. The supernatant was collected and further centrifuged at 20,000x g for 10 min at 4°C. A 50% slurry of Glutathione Sepharose 4B affinity beads (100 ml, GE Healthcare Bio-Sciences AB) equilibrated with PBS containing 10% glycerol, 0.5% Triton X-100, and 1 mM DTT (PBS-GT), was added to the final supernatant and incubated for 4 h at 4°C under gentle rotation. After incubation, affinity beads were collected by centrifugation at 780x g for 3 min and washed three times with 1 ml PBS-GT at 2300x g for 3 min at 4°C. After the final wash, affinity beads and residual buffer adjusted to 60 ml were dissolved in 60 ml of 2x sodium dodecyl sulfate (SDS) sample buffer containing 0.25 M Tris-HCl (pH 6.8), 2% SDS, 50% glycerol, and 10% 2-mercaptoethanol, and a 10-ml aliquot was subjected to SDS-polyacrylamide gel electrophoresis (7.3% polyacrylamide gel). The GST-IP_3_-sponge trapped by affinity beads was analyzed by immunoblotting. Separated proteins were transferred to an Immobilon-P transfer membrane (Millipore Corporation) and blocked with 5% skim milk in 20 mM Tris-buffered saline (pH 7.3) supplemented with 0.05% Tween 20 for 1 h. After blocking, the membrane was incubated with goat anti-GST polyclonal antibody (1:1000; Amersham Biosciences, UK Limited) overnight at 4°C and horseradish peroxidase-labeled donkey anti-goat IgG (1:5000; Jackson ImmunoResearch Laboratories, Inc.) secondary antibody for 1 h at room temperature. Signal detection was accomplished using enhanced chemiluminescent Western blotting detection reagents (Santa Cruz Biotechnology, Inc.).

**Quantification of GST-IP_3_ sponge expression**

To quantify GST-IP_3_ sponge expression in the mouse brain, a recombinant GST-IP_3_ sponge (G224/R441Q) expressed in *Escherichia coli* cells [3] was used as the quantitative standard. The plasmid vector pGEX-2T (Amersham Biosciences) carrying G224/R441Q was transformed into BL21 competent cells and expression of recombinant protein was carried out according to the method described previously [4]. The cell pellet from 50 ml culture was resuspended in 10 ml of sonication buffer containing 50 mM Tris-HCl (pH 8.0), 50 mM NaCl, 1 mM EDTA, 1 mM DTT, and protease inhibitor cocktail; treated by 5 cycles of freezing/thawing in liquid nitrogen and water bath at 37°C; and sonicated six times for 10 s each at 4°C using a VP-15s sonicator (TAITEC Co. Ltd.). After the addition of 1 ml of 10% Triton X-100 to the sonicated lysate and thorough mixing, the lysate was centrifuged at 20,000x g for 20 min at 4°C. The resulting supernatant was mixed with 150 ml of a 50% slurry of Glutathione Sepharose 4B affinity beads equilibrated with PBS-GT and incubated for 40 min at 4°C under gentle rotation. After incubation, the affinity beads were collected by centrifugation at 2000x g for 5 min at 4°C and washed three times with 4 ml PBS-GT. After the final wash, the volume of affinity beads was adjusted to 100 ml and mixed with an equal volume of 16 mM reduced glutathione dissolved in sonication buffer, and the GST-IP_3_ sponge was eluted. The protein concentration of the GST-IP_3_-sponge in the elution was determined by separating the protein using SDS-polyacrylamide gel electrophoresis (7.3%) and Coomassie Brilliant Blue gel staining applied on the twofold serially diluted elution (starting at 5 ml of the original elution up to a 128x dilution) using bovine serum albumin as a reference (range: 20 x to 0.078 mg).

GST-IP_3_ sponge levels in the mouse brain were quantified by glutathione-trapping. First, the expression of the GST-IP_3_ sponge in the brains of 3-mo-old WT, *Tg1*, and DTg mice supplied with or without Dox water and DTg mice supplied with Dox water beginning at 1 mo after birth was analyzed. The changes in GST-IP_3_-sponge expression over time were analyzed in DTg mice supplied with Dox water beginning at 1 mo after birth (3, 5, and 9-mo-old) or with a normal water supply (1, 3, 5, and 9-mo-old). To generate standard curves, the diluted recombinant GST-IP_3_ sponge was mixed with supernatant prepared from brain homogenate of C57BL/6J mice and involved the same process as for glutathione-trapping. The intensity of the signal bands in immunoblotting was used to quantify the expression level of the GST-IP_3_ sponge.

**Estimation of the GST-IP_3_-sponge number in a single astrocyte in DTg mice**

To roughly estimate the number of GST-IP_3_-sponge molecules expressed in a single astrocyte (*N*), we used the following equation:


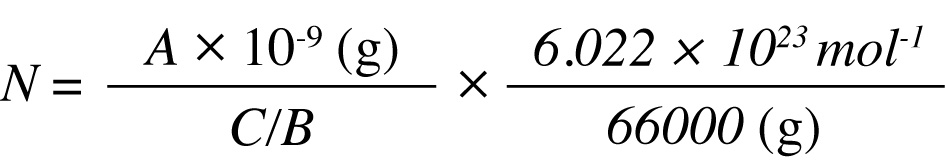


, where

*A*: GST-IP_3_-sponge expression in picograms per milligram brain tissue

*B*: Approximate specific gravity of brain tissue

*C*: Mean number of β-galactosidase-positive astrocytes per cm^3^ brain tissue

*66,000*: Molecular weight of GST-IP_3_-sponge

*6.022 x 10^23^ mol^-1^*: Avogadro constant.

Biochemical quantification of GST-IP_3_-sponge revealed that there is ~6.5 pg of GST-IP_3_-sponge per mg brain tissue in DTg mice drinking the normal water supply (Additional file 4: Fig. S2). The approximate specific gravity of mouse brain tissue is 1.047, as measured by Nelson [5]. We counted the number of S100B-positive cells in 20-µm thick frozen sections in the somatosensory cortex (SC), hippocampal CA1 (CA1), dentate gyrus (DG), and amygdala (Am) in DTg mice and the overall mean number of cells was 385 ± 65. Approximately 88% of S100B-positive cells were β-galactosidase-positive in double immunofluorescent staining. Thus, we estimated the mean cell number of β-galactosidase-positive astrocytes per cm^3^ brain tissue to be 1.41−1.98 x 10^7^ cells. When the data obtained from biochemical and histochemical examinations were extrapolated to the equation with consideration of the molecular weight of the GST-IP_3_-sponge (66,000) and Avogadro constant (6.022 x 10^23^ mol^-1^), *N* was calculated as ranging from 3136 to 4410 molecules. When the DTg mice drank Dox-containing water beginning 1 mo after birth, the expression levels of the GST-IP_3_ sponge decreased to ~0.3 pg (2 mo of Dox administration) and ~0.5 pg (8 mo of Dox administration) per mg brain tissue, suggesting that 157 to 368 molecules were expressed in a single astrocyte.

**Electrophysiology**

*Slice preparation:* The experiments were conducted with 6- to 7-mo-old male mice. All experiments were performed by an investigator blind to the mouse genotype. Mice were deeply anesthetized with halothane and subjected to cervical dislocation. The brains were immediately removed and placed in an ice-cold cutting solution containing (in mM) sucrose 200, KCl 3, NaH_2_PO_4_ 1, MgSO_4_ 10, CaCl_2_ 0.2, NaHCO_3_ 26, and D-glucose 10, saturated with 95% O_2_ and 5% CO_2_. Transverse hippocampal slices (300-µm thick) were prepared from both hemispheres. The slices were incubated in the recording solution [containing (in mM) NaCl 120, KCl 3, NaH_2_PO_4_ 1, MgSO_4_ 1.25, CaCl_2_ 2.5, NaHCO_3_ 26, and D-glucose 10, saturated with 95% O_2_ and 5% CO_2_] at room temperature (25ºC) for at least 1 h before recording.

*Extracellular recordings:* Extracellular field excitatory postsynaptic potentials (fEPSP) were recorded form the *stratum radiatum* in area CA1, filtered through a 0.1 to 10 kHz bandpass filter, and recorded at a 20-kHz sampling rate using an array of 64 planar microelectrodes (MED64) arranged in an 8x8 pattern with interelectrode spacing of 150 µm (Alpha Med Science & Co.) as described previously [6]. The stimulation comprised bipolar constant current pulses (5-20 µA, 0.1 ms duration). Input-Output curves were generated by stepping the stimulation amplitude from 5 to 19 µA. Test stimuli with the same intensity were delivered four times with 20-s interstimulus intervals and the mean fEPSP and presynaptic fiber volleys were measured. The initial slope of the fEPSP was plotted against the amplitude of the presynaptic fiber volley. For paired-pulse facilitation (PPF), the stimulation intensities were chosen to produce a fEPSP with an amplitude 30% that of the maximal fEPSP amplitude. The PPF protocol included 8 pairs of pulses with increasing intervals from 15 to 400 ms. The percentage of facilitation was calculated by dividing the fEPSP slope elicited by the second pulse by the fEPSP slope elicited with the first pulse. All data were acquired and analyzed with a MED64 Mobius (Alpha Med Science & Co.).

**Behavioral analysis**

All experiments were performed by an investigator blind to the mouse genotype.

*Open field test*: The experiments were conducted with 3-mo-old male mice. Locomotor activity in a novel environment was measured in a white open field apparatus (50 x 50 cm) illuminated at 70 lux (surface level of the arena). Each mouse was placed in the center of the arena, and horizontal activity was monitored for 25 min with a video camera and processed with NIH Image OF software (O'Hara &Co.). The center of the open field was defined as the central 20% of the total area.

*Elevated plus maze test*: The experiments were conducted with 3.5-mo-old male mice. The apparatus comprised two open arms (25 x 5 cm) and two closed arms of the same size with 15-cm high transparent walls (O'Hara &Co.). The maze was elevated to a height of 50 cm above the floor and illuminated at 200 lux (surface of open arms). Each mouse was placed in the central square of the maze, facing one of the open arms. Behavior was monitored for 10 min with a video camera and images were processed with NIH Image EP software (O'Hara &Co.).

*Home cage activity:* The experiments were conducted with 6- to 7-mo-old male mice. To measure locomotor activity, each mouse was placed in a transparent cage (20.7 [W] x 36.5 [L] x 14 [H] cm) with bedding, and vertical and horizontal activity was measured for 7 days using infrared sensors (Scanet; Melquest, Toyama, Japan).

**References**

1. Mayford M, Bach ME, Huang YY, Wang L, Hawkins RD, Kandel ER: **Control of memory formation through regulated expression of a CaMKII transgene.** *Science* 1996, **274:**1678-1683.

2. Tanaka M, Yamaguchi K, Tatsukawa T, Nishioka C, Nishiyama H, Theis M, Willecke K, Itohara S: **Lack of Connexin43-mediated bergmann glial gap junctional coupling does not affect cerebellar long-term depression, motor coordination, or eyeblink conditioning.** *Front Behav Neurosci* 2008, **2:**1.

3. Uchiyama T, Yoshikawa F, Hishida A, Furuichi T, Mikoshiba K: **A novel recombinant hyperaffinity inositol 1,4,5-trisphosphate (IP(3)) absorbent traps IP(3), resulting in specific inhibition of IP(3)-mediated calcium signaling.** *J Biol Chem* 2002, **277:**8106-8113.

4. Yoshikawa F, Uchiyama T, Iwasaki H, Tomomori-Satoh C, Tanaka T, Furuichi T, Mikoshiba K: **High efficient expression of the functional ligand binding site of the inositol 1,4,5-triphosphate receptor in Escherichia coli.** *Biochem Biophys Res Commun* 1999, **257:**792-797.

5. Nelson SR: **Effect of drugs on experimental brain edema in mice.** *J Neurosurg* 1974, **41:**193-199.

6. Sano Y, Ornthanalai VG, Yamada K, Homma C, Suzuki H, Suzuki T, Murphy NP, Itohara S: **X11-like protein deficiency is associated with impaired conflict resolution in mice.** *J Neurosci* 2009, **29:**5884-5896.
